# Metabolomic profiling of the purple sulfur bacterium *Allochromatium vinosum* during growth on different reduced sulfur compounds and malate

**DOI:** 10.1007/s11306-014-0649-7

**Published:** 2014-05-22

**Authors:** Thomas Weissgerber, Mutsumi Watanabe, Rainer Hoefgen, Christiane Dahl

**Affiliations:** 1 Institut für Mikrobiologie & Biotechnologie, Rheinische Friedrich-Wilhelms-Universität Bonn, Meckenheimer Allee 168, 53115 Bonn, Germany; 2 Max-Planck-Institut für Molekulare Pflanzenphysiologie, Science Park Potsdam – Golm, 14424 Potsdam, Germany

**Keywords:** *Allochromatium vinosum*, Metabolomic profiling, Purple sulfur bacteria, Sulfur oxidation, Assimilatory sulfate reduction

## Abstract

Environmental fluctuations require rapid adjustment of the physiology of bacteria. Anoxygenic phototrophic purple sulfur bacteria, like *Allochromatium vinosum*, thrive in environments that are characterized by steep gradients of important nutrients for these organisms, i.e., reduced sulfur compounds, light, oxygen and carbon sources. Changing conditions necessitate changes on every level of the underlying cellular and molecular network. Thus far, two global analyses of *A. vinosum* responses to changes of nutritional conditions have been performed and these focused on gene expression and protein levels. Here, we provide a study on metabolite composition and relate it with transcriptional and proteomic profiling data to provide a more comprehensive insight on the systems level adjustment to available nutrients. We identified 131 individual metabolites and compared availability and concentration under four different growth conditions (sulfide, thiosulfate, elemental sulfur, and malate) and on sulfide for a Δ*dsrJ* mutant strain. During growth on malate, cysteine was identified to be the least abundant amino acid. Concentrations of the metabolite classes “amino acids” and “organic acids” (i.e., pyruvate and its derivatives) were higher on malate than on reduced sulfur compounds by at least 20 and 50 %, respectively. Similar observations were made for metabolites assigned to anabolism of glucose. Growth on sulfur compounds led to enhanced concentrations of sulfur containing metabolites, while other cell constituents remained unaffected or decreased. Incapability of sulfur globule oxidation of the mutant strain was reflected by a low energy level of the cell and consequently reduced levels of amino acids (40 %) and sugars (65 %).

**Electronic supplementary material:**

The online version of this article (doi:10.1007/s11306-014-0649-7) contains supplementary material, which is available to authorized users.

## Introduction

The adaption of biological systems to changes in their environment is characterized by immediate and appropriate adjustment of physiology on every level of the cellular and molecular network. Responses on the level of the transcriptome are transient and result in a subsequent new steady state on the proteome and metabolome levels. The purple sulfur bacterium *Allochromatium vinosum* DSM 180^T^, a member of the family *Chromatiaceae* within the gamma class of the phylum *Proteobacteria*, is one of the best studied anoxygenic phototrophic bacteria. It is not only capable of photolithoautotrophic growth on reduced sulfur compounds (sulfide, polysulfide, thiosulfate, elemental sulfur) fixing CO_2_ as a carbon source, but can also grow as a photoorganoheterotroph on organic acids, like malate (Imhoff 2005; Weissgerber et al. 2011). Sunlight is the primary energy source, while electrons are obtained from reduced sulfur compounds or organic acids. An understanding of the biological processes involved in sulfur oxidation is of major interest, since purple sulfur bacteria flourish wherever light reaches sulfidic water layers or sediments and often occur as dense accumulations in conspicuous blooms in freshwater as well as in marine aquatic ecosystems. Here, they are major players in the reoxidation of sulfide produced by sulfate-reducing bacteria in deeper anoxic layers.

**Fig. 1:**
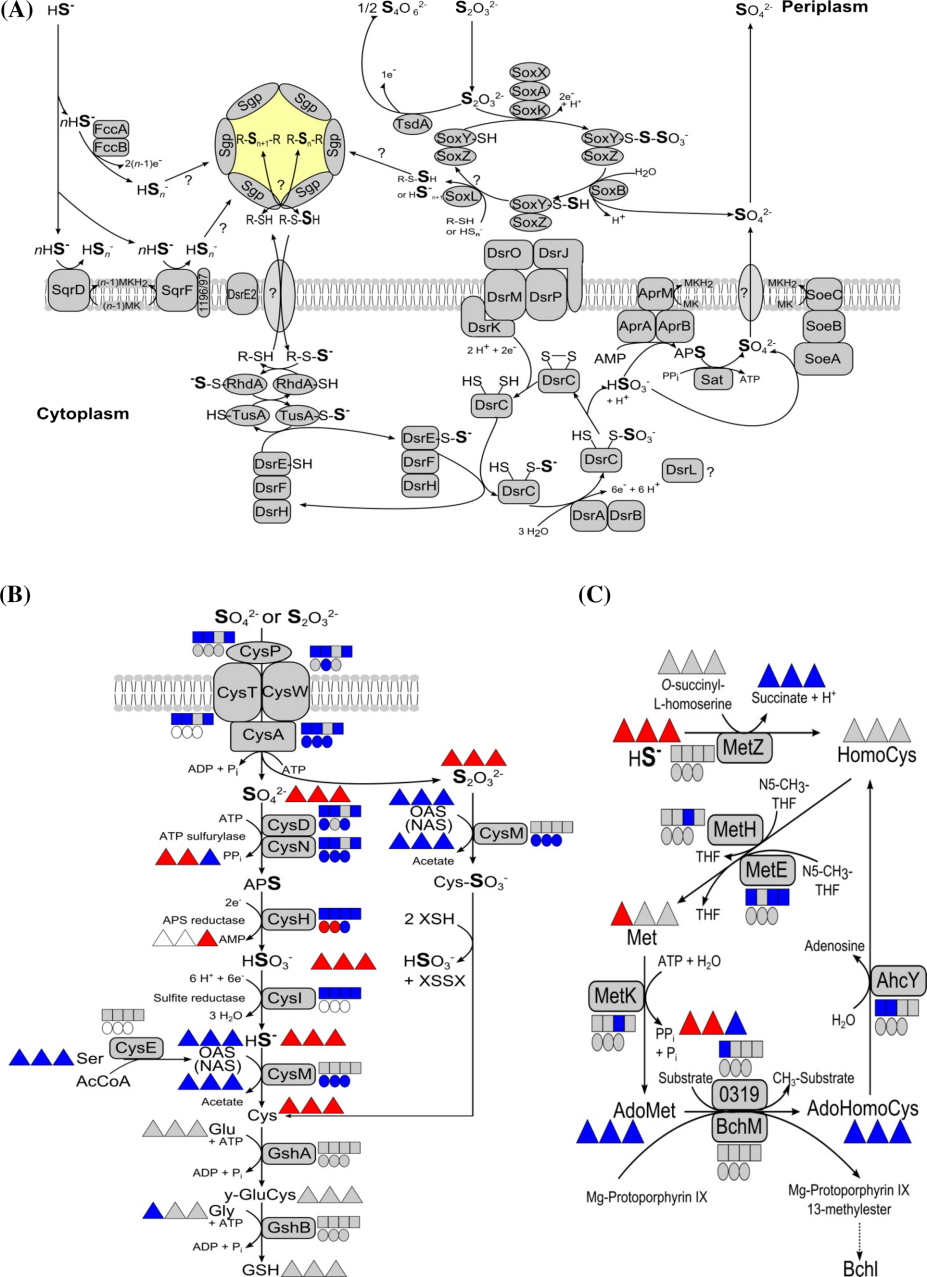
Current models of dissimilatory sulfur oxidation (**a**), assimilatory sulfate reduction, cysteine and glutathione biosynthesis (**b**) as well as methionine biosynthesis and methylation reactions (**c**) in *Allochromatium vinosum*. **a** Polysulfides are the first products of sulfide oxidation. Polysulfur chains (HS_*n*_^−^) in the periplasm are probably very short (*n* probably around 3 or 4), whereas the polysulfur chains in the sulfur globules can be very long (*n* > 3 and possibly up to *n* > 10^5^ as for polymeric sulfur) (Dahl and Prange 2006; Prange et al. 2002). Transport of sulfane sulfur into the cytoplasm is proposed to proceed via a low molecular weight carrier molecule, possibly glutathione (amide). The carrier molecule is indicated as “RSH”. Sulfite is formed in the cytoplasm by the enzymes of the Dsr (dissimilatory sulfite reductase) system. *Sgp* sulfur globule proteins, *FccAB* flavocytochrome *c*, *Sqr* sulfide:quinone oxidoreductase, *TsdA* thiosulfate dehydrogenase, *Sox* periplasmic thiosulfate oxidizing multienzyme complex, *Rhd* rhodanese-like protein, *Apr* adenosine-5′-phosphosulfate reductase, *Sat* dissimilatory ATP sulfurylase, *Soe* sulfite oxidizing enzyme. **b** Assimilatory sulfate reduction in *A. vinosum* does not involve formation of phosphoadenosine-5′-phosphosulfate (Neumann et al. 2000). *CysE* serine *O*-acetyltransferase (Alvin_0863), *CysM* cysteine synthase B (Alvin_2228), *GshA* glutamate/cysteine ligase (Alvin_800), *GshB* glutathione synthetase (Alvin_0197), *γ-GluCys* γ-glutamylcysteine, *GSH* glutathione, *XSH* glutathione, reduced thioredoxin or glutaredoxin, *XSSX* oxidized glutathione, thioredoxin or glutaredoxin (see text for further explanation), *OAS* *O*-acetyl-serine, *NAS* *N*-acetyl-serine, *Cys-SO*_*3*_^*−*^ *S*-sulfocysteine. **c** Biosynthesis of homocysteine (HomoCys), methionine and biological methylation in *A. vinosum*. *AdoMet* *S*-adenosylmethionine, *AdoHomoCys* *S*-adenosylhomocysteine, *N5-CH*_*3*_ *-THF* N5-methyl-5,6,7,8-tetrahydrofolate, *MetZ* *O*-succinyl-l-homoserine sulfhydrylase (Alvin_1027), *MetE* cobalamin-independent methionine synthase (Alvin_2262), *MetH* cobalamin-dependent methionine synthase (Alvin_1622), *AhcY* adenosylhomocysteinase (Alvin_0320), *BchM* magnesium protoporphyrin *O*-methyltransferase (Alvin_2638), *MetK* *S*-adenosylmethionine synthetase (Alvin_0318); 0319, methyltransferase type 11 (Alvin_0319). The transcriptomic (*boxes*) (Weissgerber et al. 2013), proteomic (*circles*) (Weissgerber et al. 2014) and metabolomic profiles (*triangles*) (all relative to growth on malate) are depicted next to the respective protein/metabolite. Relative fold changes in mRNA levels above 2 (*red*) were considered significantly enhanced. Relative changes smaller than 0.5 (*blue*) were considered as indicating significant decreases in mRNA levels. Relative fold changes between 0.5 and 2 (*grey*) indicated unchanged mRNA levels. The same color coding is applied to changes on the protein and metabolome levels. Here, values above 1.5 (*red*) and below 0.67 (*blue*) were considered significant. Those cases, where transcriptomic data was not available or the respective protein or metabolite was not detected in the proteomic or metabolomic approach, respectively, are indicated by *white squares*, *circles* or *triangles*. Sulfur compounds added from left to right: sulfide, thiosulfate, elemental sulfur and sulfite. Changes on sulfite were not determined on the proteome and metabolome levels

In *A. vinosum*, sulfur compounds, such as sulfide, polysulfides, elemental sulfur or thiosulfate, enter the sulfur oxidation pathway via the formation of sulfur globules (Frigaard and Dahl 2009). These globules are located in the bacterial periplasm (Pattaragulwanit et al. 1998) and result in a milky appearance of the cells. According to the current model (Fig. 1a), sulfide oxidation is catalyzed by at least three periplasmically oriented enzymes, namely the soluble flavocytochrome *c* and the membrane-bound sulfide:quinone-oxidoreductases SqrD and SqrF (Gregersen et al. 2011; Reinartz et al. 1998; Weissgerber et al. 2011). The oxidation of thiosulfate is mediated by the Sox proteins SoxYZ, SoxB, SoxXAK and SoxL resulting in formation of sulfate (Hensen et al. 2006; Welte et al. 2009) whilst the diheme cytochrome *c* thiosulfate dehydrogenase catalyzes the formation of tetrathionate as final product. The latter reaction is favored under slightly acidic conditions (Denkmann et al. 2012; Hensen et al. 2006). Oxidation of the sulfur stored in the globules to sulfite is catalyzed by the Dsr system including dissimilatory sulfite reductase (DsrAB) (Dahl et al. 2005; Lübbe et al. 2006; Pott and Dahl 1998; Sander et al. 2006). Most proteins of the Dsr system are absolutely essential for degradation of sulfur globules. These include the triheme cytochrome *c* DsrJ, a component of the electron-transporting transmembrane complex DsrMKJOP (Grein et al. 2010; Sander et al. 2006). The oxidation of sulfite, the product of the Dsr pathway, to sulfate is performed either indirectly via adenosine-5′-phosphosulfate (APS) catalyzed by APS reductase and ATP sulfurylase or directly via the cytoplasmically oriented membrane-bound iron–sulfur molybdoenzyme SoeABC (Dahl et al. 2013). The processes occurring during uptake and oxidation of externally supplied elemental sulfur by *A. vinosum* and other purple sulfur bacteria are not well understood (Franz et al. 2007). It has been firmly established that direct physical contact between elemental sulfur and the *A. vinosum* cell surface is of essential importance for elemental sulfur oxidation (Franz et al. 2007). It is not known, whether specific outer membrane proteins or production of glycocalyx-like material may be involved as has been documented for some chemotrophic sulfur oxidizers (Bryant et al. 1984). In absence of reduced sulfur compounds, cell requirement for sulfur in cell components, e. g. cysteine, is satisfied by assimilatory sulfate reduction (Fig. 1b) (Neumann et al. 2000).

In contrast to plants, metabolome analyses on prokaryotes are still rare. Most of the few available studies were performed with *Escherichia coli* (e.g. Bennett et al. 2009; Jozefczuk et al. 2010), some with cyanobacteria (e.g. Eisenhut et al. 2008) or with *Staphylococcus aureus* (Sun et al. 2012). To our knowledge, there is no study available concerning metabolites present in *A. vinosum* or any other anoxygenic phototrophic sulfur bacterium. Recently, the complete *A. vinosum* genome sequence was analyzed (Weissgerber et al. 2011) and global transcriptomic and proteomic analyses were performed, that compared autotrophic growth on different reduced sulfur sources with heterotrophic growth on malate (Weissgerber et al. 2013, 2014). Thus, global analyses of the *A. vinosum* response to nutritional changes so far have been limited to two levels of information processing, namely transcription and translation. A similar approach on the metabolome level is clearly missing to apprehend the system in its whole. Specifically, comprehensive analysis of changes on the level of metabolites can be regarded as a promising approach not only for a first glimpse into systems biology of anoxygenic phototrophs, but possibly also for answering open questions regarding dissimilatory sulfur metabolism. We therefore set out to analyze the metabolomic patterns of *A. vinosum* wild type during growth on malate and the reduced sulfur compounds sulfide, thiosulfate and elemental sulfur. To complete the picture, we also evaluated the metabolomic patterns of the sulfur oxidation deficient *A. vinosum* Δ*dsrJ* strain during growth on sulfide. Experiments were designed such that they enabled integration of metabolic, proteomic and transcript changes under the four different growth conditions. The resulting data sets allowed us to identify parallel and distinct response patterns, represented by conserved patterns on both the metabolic and the gene and protein expression levels, across all sulfur compounds.

## Materials and methods

### Bacterial strains, plasmids and growth conditions

Bacterial strains used in this study were *A. vinosum* Rif50, a spontaneous rifampicin-resistant mutant of the wild type strain *A. vinosum* DSM 180^T^ (Lübbe et al. 2006), and the corresponding Δ*dsrJ* mutant strain (Sander et al. 2006). Cells grown photoorganoheterotrophically on malate (RCV medium (Weaver et al. 1975)) for 3 days were used as an inoculum for metabolome experiments. The culture volume of the precultures was 1,000 ml. Inoculum cells were harvested by centrifugation (10 min, 2,680×*g*), washed once in modified Pfennig′s medium (“0” medium without sulfide) (Hensen et al. 2006) and transferred to 250 ml culture bottles. To guarantee comparable starting cell densities (OD_690_ = 0.9), the optical density at 690 nm of the precultures was determined and the necessary volume for inoculation was exactly calculated. For metabolome experiments, the cells were then cultivated photolithoautotrophically in batch culture at 30 °C under anoxic conditions and continuous illumination in completely filled, stirred screw-capped 250-ml culture bottles containing “0” medium. Concentration of ammonium chloride was set to 1.2 g l^−1^ in all cases. Sulfide (4 mM), thiosulfate (10 mM) or 50 mM elemental sulfur [obtained from Riedel-de Haën, consisting of 30 % cyclo-octasulfur and 70 % polymeric sulfur (Franz et al. 2009b)] were added to the cultures as sulfur sources. For photoorganoheterotrohic growth on malate with sulfate as sole sulfur source, “0” medium was mixed with 22 mM malate (pH 7.0 of malate stock solution was reached by the addition of NaOH). Incubation times prior to sample collection were set as follows: 8 h for growth on sulfide, thiosulfate and malate. When elemental sulfur was the substrate, incubation was prolonged to 24 h. Experiments were performed with five biological replicates for each substrate. Growth conditions and sampling points were exactly the same in a comparative quantitative proteome study on *A. vinosum* (Weissgerber et al. 2014). Growth conditions were also identical for global transcriptomic profiling, however, incubation times after addition of substrates were shorter in this case (1, 2 and 3 h hours on sulfide, thiosulfate and elemental sulfur, respectively). This was necessary because transcriptomic responses occur earlier in time and proved to be only transient in many cases. With regard to the pathways of central carbon metabolism, hydrogen metabolism as well as dissimilatory sulfur oxidation and assimilatory sulfate reduction, the transcriptomic and proteomic responses matched in most instances substantiating the incubation times as well chosen (Weissgerber et al. 2014). Rifampicin was used in a final concentration of 50 μg ml^−1^ for the precultures. Protein concentrations were determined as described previously (Franz et al. 2007).

### Measurement of primary metabolites by GC–TOF–MS analysis

10 ml culture was filtered through cellulose nitrate filters of 0.45 μm pore size and 2.5 cm diameter. The filtrates were extracted in 600 μl methanol at 70 °C for 15 min and then 400 μl of chloroform at 37 °C for 5 min. The polar fraction was prepared by liquid partitioning into 800 μl of water (ULC/MS grade). The polar fraction (300 μl) was evaporated and then derivatized by methoxyamination and subsequent trimethylsilylation. Samples were analyzed by GC–TOF–MS (ChromaTOF software, Pegasus driver 1.61, LECO, St Joseph, MI, USA). GC-TOF–MS analysis was performed as previously described (Erban et al. 2007; Lisec et al. 2006). The chromatograms and mass spectra were evaluated using the TagFinder software (Luedemann et al. 2008) and NIST05 software (http://www.nist.gov/srd/mslist.htm). Metabolite identification was manually supervised using the mass spectral and retention index collection of the Golm Metabolome Database (Hummel et al. 2010; Kopka et al. 2005). Peak heights of the mass fragments were normalized on the added amount of an internal standard (^13^C_6_-sorbitol).

### Measurement of ion contents

The polar fraction (200 μl) from GC–TOF–MS extraction was evaporated and then dissolved in 550 μl of water (ULC/MS grade). Samples were analyzed by Dionex ICS-3000 system with a KOH gradient for anions and with a methanesulfonic acid gradient for cations.

### Measurement of thiol contents

Measurement of thiols was performed by a combination of monobromobimane fluorescent labeling and HPLC (Anderson 1985; Fahey and Newton 1987). The polar fraction (200 μl) from GC–TOF–MS extraction was evaporated and then dissolved in 60 μl of 0.1 M HCl. A mixture of 20 μl of the extract and 40 μl of 25 μM *N*-acetyl-cysteine as a internal standard was reacted with 3 μl of 30 mM tris(2-carboxyethyl)phosphine as a reducing reagent and 10 μl of 8.5 mM *N*-ethylmorpholine buffer at 37 °C for 20 min. The total thiols were derivatized by the addition of 3 μl of 30 mM monobromobimane at 37 °C for 20 min in dark. The labeling reaction was terminated by the addition of 10 μl of acetic acid and the resulting solution was then subjected to HPLC analysis. HPLC was carried out as described previously (Saito et al. 1994).

### Measurement of adenosine derivatives

Adenosine derivatives were quantified fluorometrically after specific derivatization of adenosine compounds with chloroacetaldehyde (CAA) based on a method previously described (Bürstenbinder et al. 2007). The polar fraction (200 μl) from GC–TOF–MS extraction was evaporated and then dissolved in 15 μl of 0.1 M HCl. The extract (15 μl) mixed with 77 μl of CP buffer [62 mM citric acid-1-hydrate and 76 mM (Na)_2_HPO_4_·2H_2_O, pH 4] was derivatized by adding 8 μl of 45 % (v/v) chloroacetaldehyde for 10 min at 80 °C. The analyses of adenosines was performed by reverse-phase HPLC on a Hyperclone C18 (ODS) column (Phenomenex, Aschaffenburg, Germany) connected to an HPLC system (Dionex). The HPLC analysis was carried out as described previously (Estavillo et al. 2011).

### Measurement of amino acid contents

The polar fraction (200 μl) from GC–TOF–MS extraction was evaporated and then dissolved in 60 μl of 0.1 M HCl. The extracts (30 μl) were subjected to HPLC analysis using a Hyperclone C18 (ODS) column (Phenomenex, Aschaffenburg, Germany) connected to an HPLC system (Dionex). Amino acids were determined by pre-column online derivatization with *O*-phthalaldehyde in combination with fluorescence detection (Kim et al. 1997; Lindroth and Mopper 1979).

### Statistics

*p* values were calculated by a paired, two tail Student’s t test (Excel, Microsoft Office). For the wild type relative concentration of each metabolite after growth on each sulfur compound was compared with that after growth on malate. For the metabolite concentrations of the ∆*dsrJ* mutant strain on sulfide comparison was drawn to wild type metabolites after growth on sulfide.

## Results and discussion

### Experimental design

An established metabolic profiling platform was used to characterize the metabolic response of *A. vinosum* to four different growth conditions, comprising photolithoautotrophic growth on sulfide, thiosulfate, elemental sulfur and photoorganoheterotrophic growth on malate. Each experimental condition was independently repeated five times. For the analysis of the metabolomic patterns of *A. vinosum*, cells were grown photoorganoheterotrophically on 22 mM malate (8 h) or photolithoautotrophically on 4 mM sulfide (8 h), 10 mM thiosulfate (8 h) or 50 mM elemental sulfur (24 h), respectively. The experiments were designed such that effects exerted by different growth rates and different cell densities were minimized: The incubation periods chosen correspond to those, after which *A. vinosum* exhibits maximum stable sulfate production rates (Weissgerber et al. 2014). It should be noted, that during growth on 4 mM sulfide, extracellular sulfide is depleted ca 4 h after inoculation (Dahl et al. 2013). Hence, whilst sulfide was the originally provided substrate, metabolic analysis was performed with cells that had already started to oxidize intracellularly stored sulfur reserves. Starting optical densities (OD690: ~0.9) and protein contents (0.10 ± 0.01 mg ml^−1^) were identical for all cultures. Appreciable growth of the cells had not occurred in any of the cultures at the time of metabolite analysis. Protein concentrations (in mg ml^−1^) at this time point were virtually identical in all cases: 0.10 ± 0.01 on malate, 0.11 ± 0.00 on sulfide; 0.11 ± 0.00 on thiosulfate, 0.12 ± 0.00 on elemental sulfur, and 0.10 ± 0.00 for ∆*dsrJ* on sulfide. The experiments were designed both to compare metabolic changes imparted by changing electron donors (malate and different sulfur compounds) and carbon sources (malate versus CO_2_) for biosynthesis of cellular carbon constituents._._In order to investigate possible metabolic changes in a mutant incapable of oxidizing sulfur stored in periplasmic sulfur globules, we also performed an experiment with a Δ*dsrJ* mutant strain (Sander et al. 2006) on sulfide.

In total, 131 individual metabolites were detected (Fig. S1; Table S1). Besides sulfur compounds (hydrogen sulfide, thiosulfate, sulfite) and glutathione intermediates, these comprise among others major components of glycolysis/gluconeogenesis, the citric acid cycle and all standard amino acids except proline. In addition, we detected major products of fatty acid biosynthesis, several important cations (e.g. ammonium), anions (e.g. sulfate) and indicators for the energy level of the cell. This resulted in the description of metabolite occurrence and proportions in the original state, namely photoorganoheterotrophic growth on malate, differences between growth on malate and sulfur compounds as well as on differences between the *A. vinosum* wild type and the Δ*dsrJ* mutant strain.

### Photoorganoheterotrophic growth on malate

Since the precultures were grown photoorganoheterotrophically on malate, this was defined as the basic state of the cells. In *A. vinosum*, malate enters carbon metabolism via the formation of pyruvate catalyzed by malic enzyme (Alvin_3051) (Sahl and Trüper 1980). Another possibility is the formation of oxaloacetate mediated by a malate:quinone oxidoreductase (Alvin_2732), that is predicted by the genome sequence. The high relative amounts of malic acid and pyruvic acid (Table S1) indicate formation of pyruvate as the major reaction matching earlier reports (Sahl and Trüper 1980). As a next step, pyruvate can be decarboxylated for oxidation via the citric acid cycle or converted into phosphoenolpyruvate catalyzed by Alvin_0839 (pyruvate water dikinase) or Alvin_2105 [pyruvate phosphate dikinase (Buchanan 1974)] for gluconeogenesis or regeneration of oxaloacetate via phosphoenolpyruvate carboxylase (Alvin_2986) (Fuller et al. 1961). The relative amounts of malic acid and of the citric acid cycle intermediates fumaric acid and succinic acid were found to be comparably high, probably due to the reversibility of the reactions, and the relative contents of these metabolites were apparently higher than those for the other detected citric acid cycle intermediates indicating accumulation of these metabolites (Table S1). Except for 1,3-bisphosphoglyceric acid, glyceraldehyde-3-phosphate, dihydroxyacetone-phosphate and fructose-1,6-bisphosphate, we detected all intermediates of gluconeogenesis (Table S1).

**Fig. 2:**
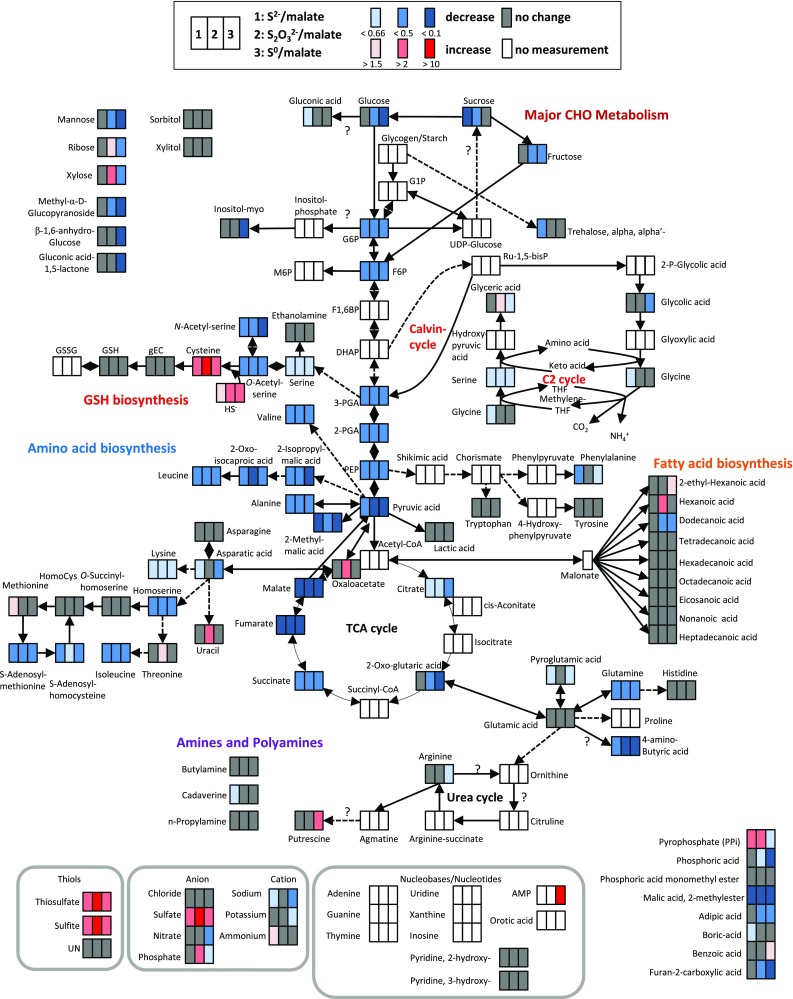
Simplified scheme of *A. vinosum* central metabolism comparing metabolite concentrations after growth on malate with those after growth on sulfide, thiosulfate and elemental sulfur. Color range visualizes changes of at least 1.5-fold, twofold and tenfold, respectively

Relative amounts of intermediates and products of amino acid anabolism revealed a complex picture. Starting from oxalic acid, the amino acids aspartate, lysine, asparagine, threonine, isoleucine and methionine are formed (Fig. 2). Aspartate is the predominating amino acid within this family, because aspartate kinase is feedback inhibited by lysine, threonine and methionine preventing further transformation of aspartate to the other amino acids (Table S1) (Datta and Gest 1964; Truffa-Bachi and Cohen 1968; Umbarger 1969). Isoleucine is the least abundant representative of aspartic acid family. 2-Oxo-glutaric acid is the precursor for glutamate, glutamine, proline and arginine (Fig. 2). Noteworthy, glutamic acid (16 nmol mg^−1^ protein) and aspartic acid (12 nmol mg^−1^ protein) are the dominating proteinogenic amino acids in *A. vinosum* (Table S1). The pyruvic acid amino acid family comprises alanine, valine, leucine and isoleucine (Fig. 2). Within this group, alanine predominates (Table S1). Transformation of 3-phosphoglyceric acid can result in the synthesis of the amino acids serine, glycine and cysteine (Fig. 2). Here, serine (0.8 nmol mg^−1^ protein) is the first intermediate. Concentrations of its derivatives glycine (0.2 nmol mg^−1^ protein) and cysteine (0.04 nmol mg^−1^ protein) were significantly lower (Table S1). Drawing correlations between glycine and other amino acids of the 3-phosphoglyceric acid family is difficult, because glycine can be produced both from serine by a glycine hydroxymethyltransferase reaction and from glyoxylate by a transaminase reaction in *A. vinosum*. These reactions are part of the plant-like C2 glycolate cycle for photorespiration described for the cyanobacterium *Synechocystis* sp. (Eisenhut et al. 2006). Corresponding genes (Alvin_0271, _1931, _0550, _1774 and _2085) are also present in *A. vinosum* and their transcripts and proteins were detected (Weissgerber et al. 2013, 2014). The aromatic amino acids tyrosine, phenylalanine and tryptophan require the precursors phosphoenolpyruvate (Fig. 2) and erythrose-4-phosphate for their synthesis and share seven initial reaction steps. Here, tyrosine predominates (Table S1). Notably, the sulfur containing amino acid cysteine represents the least abundant amino acid in the cell during growth on malate (Fig. 2; Table S1).

Determination of fatty acids revealed the presence of compounds with chain lengths of 6, 9, 12, 14, 16, 17 and 20 carbon atoms in *A. vinosum* cells (Table S1).

### Photoorganoheterotrophic growth on malate versus photolithoautotrophic growth on sulfur compounds (wild type)

**Fig. 3:**
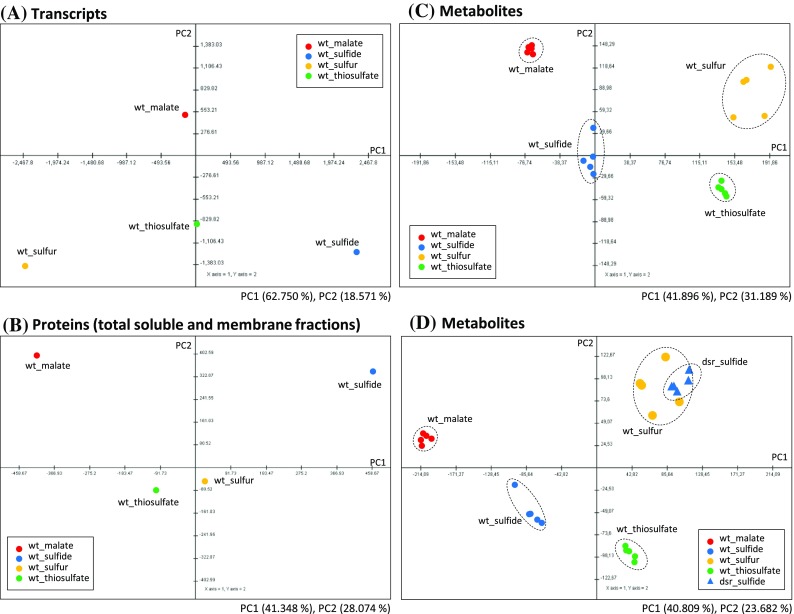
Principal component analysis (PCA) score plot of transcript data (**a**) protein data (**b**) and metabolite data (**c**) for *A. vinosum* wild type. The plots were applied for the 3,271 genes, 1,876 proteins and the 131 metabolites. The average data from 3 to 4 biological replications and 2 biological replications, which were previously published (Weissgerber et al. 2013, 2014) were used for the PCA of transcript data and protein data, respectively. **d** PCA score plot of metabolite data including *ΔdsrJ* mutant strain. The plot was applied for the 131 metabolites. PCA was conducted by the MultiExperiment Viewer (Saeed et al. 2003). *PC* principal component

A principal component analysis (PCA) of previously obtained transcriptome (Weissgerber et al. 2013) and proteome data (Weissgerber et al. 2014) and the metabolome data of this study was performed on wild type *A. vinosum* under sulfide, sulfur, thiosulfate and malate conditions (Fig. 3a–c). All three data sets are well separated from one another in the PCA score plot indicating sufficiently high differences between all four growth conditions. This is indicative for specific regulatory adaptations (Fig. 3a, b) of the system, which eventually lead to distinctively different physiological states as exemplified by the metabolome separations (Fig. 3c). PC1 separates transcriptome data in the order sulfide, thiosulfate and elemental sulfur, which corresponds to the known physiology behind exploiting these substrates, while malate data are separated from all three supplied sulfur compounds equally by PC2 indicating activation of a completely different gene set. At the proteome and metabolome level (Fig. 3b, c), the four conditions are clearly separated from one another indicating different protein and metabolite compositions, respectively, in each case. This means, that *A. vinosum* very flexibly adapts to each of the conditions reaching a distinct physiological state. On the metabolome level, PC1 and PC2 do not provide an as strictly ordered separation of the conditions as in case of the transcriptome. When growing *A. vinosum* on elemental sulfur, it displays higher variation between the experiments (each dot represents one complete experimental data set). Probably, variation is representative for the fact that exploitation of elemental sulfur depends on additional factors not fully controlled in this experiment, while the response to all other conditions is very consistent. Fitting to this, two major observations were made upon the switch from growth on malate to growth on sulfur compounds and carbon dioxide, which results in both, changes of electron donor and carbon source: Firstly, relative content of sulfur-containing metabolites increased significantly and secondly, relative amounts of gluconeogenetic/glycolytic as well as citric acid cycle intermediates decreased drastically. These data are discussed in detail below.

#### *A. vinosum* under different S regimes

**Fig. 4:**
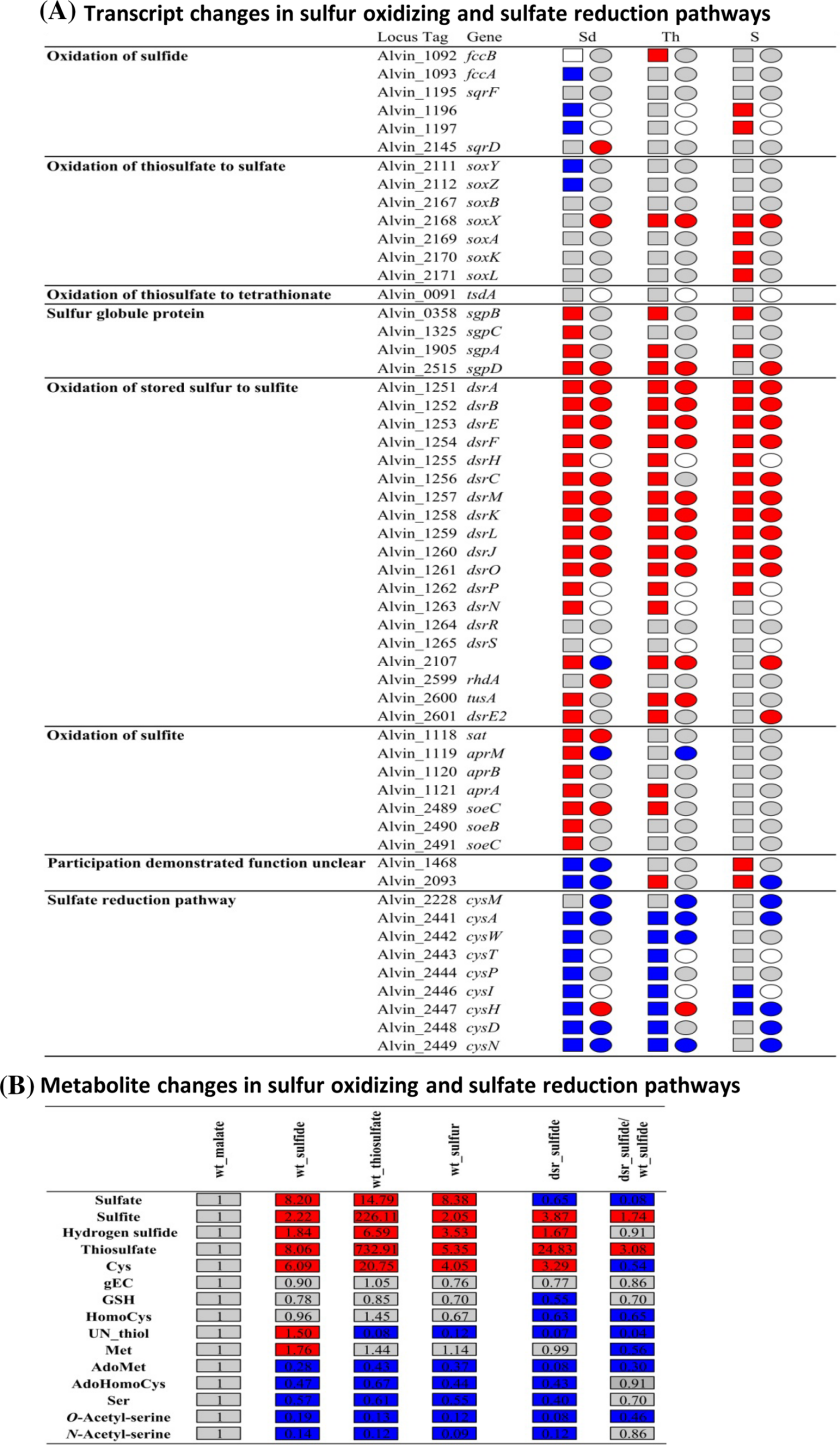
Transcript (Weissgerber et al. 2013), protein (Weissgerber et al. 2014) (**a**) and metabolite changes (**b**) in sulfur oxidizing and sulfate reduction pathways. The transcriptomic (*boxes*) (Weissgerber et al. 2013) and proteomic (*circles*) (Weissgerber et al. 2014) profiles (all relative to growth on malate) are depicted next to the respective locus tag. Relative fold changes in mRNA levels above 2 (*red*) were considered significantly enhanced. Relative changes smaller than 0.5 (*blue*) were considered as indicating significant decreases in mRNA levels. Relative fold changes between 0.5 and 2 (*grey*) indicated unchanged mRNA levels. The same color coding is applied to changes on the protein levels. Here, values above 1.5 (*red*) and below 0.67 (*blue*) were considered significant. Those cases, where transcriptomic data was not available or the respective protein not detected in the proteomic approach, respectively, are indicated by white squares or circles. *Sd* sulfide, *Th* thiosulfate, *S* elemental sulfur

It was previously reported that the presence of reduced sulfur compounds resulted in elevated relative mRNA and protein levels for genes/proteins of central enzymes of oxidative sulfur metabolism, while transcript and protein levels for genes/proteins involved in assimilatory sulfate reduction were negatively affected (Weissgerber et al. 2013, 2014) (see also Figs. 1b, 4a). These responses are positively correlated to the concentration changes of the metabolites of the affected metabolic pathways. Concentrations of the substrates sulfide and thiosulfate as well as of the intermediate sulfite, that is formed en route to sulfate, were significantly higher in sulfur-grown than in malate-grown cells (Fig. 4b). As expected, intracellular sulfate concentrations in cells grown with either one of the three different sulfur sources significantly exceeded the intracellular sulfate concentrations in malate-grown cells (Fig. 4b; Fig. S1; Table S1). While intracellular sulfate originates from complete oxidation of the provided sulfur compounds when grown photolithoautotrophically on sulfur compounds, sulfate present in malate-grown cells must have completely been taken up from the medium. Our data reveal that the intracellular concentration of cysteine is a suitable biological indicator for the availability of reduced sulfur in the cell.

Biosynthesis of cysteine requires the formation of *O*-acetyl-l-serine, which is then further transformed to cysteine catalyzed by cysteine synthase B (CysM) in a reaction that is dependent on the availability of sulfide (Fig. 1b) (Hensel and Trüper 1976). It is well established that the CysTWA ABC-type transporter in conjunction with the periplasmic binding protein CysP transports not only sulfate but also thiosulfate into the cytoplasm (Sirko et al. 1995) (Fig. 1b). In *Salmonella typhimurium* and *E. coli*, cysteine synthase B (CysM) also accepts thiosulfate as a substrate and hooks it up to *O*-acetyl-l-serine resulting in the formation of *S*-sulfocysteine (Kredich 1992). *S*-sulfocysteine is then reduced to cysteine resulting in the release of sulfite (Nakatani et al. 2012; Sekowska et al. 2000). Glutathione, thioredoxins or glutaredoxins have been discussed as possible reductants in this reaction (Funane et al. 1987; Nakatani et al. 2012; Woodin and Segel 1968). A similar reaction sequence is also probable for the assimilation of thiosulfate in *A. vinosum* (Fig. 1b). In fact, thiosulfate was previously detected intracellularly in *A. vinosum* (Franz et al. 2009a) and this was confirmed in the current study. It is noteworthy, that the intracellular concentration of sulfite is highest during growth on thiosulfate. Sulfite release from *S*-sulfocysteine as described above may contribute to the observed elevated sulfite level on this substrate.

During growth on malate, sulfide for biosynthesis of sulfur containing cell constituents is provided by the assimilatory sulfate reduction pathway in an energy consuming process (Fig. 1b) (Neumann et al. 2000), while sulfide is readily available without any input of energy under sulfur-oxidizing conditions. Correspondingly, cysteine predominates during photolithoautotrophic growth on sulfur compounds (Figs. 1b, 4b). The cysteine precursor *O*-acetyl-l-serine is transformed non-enzymatically into *N*-acetyl-serine through an *O*- to *N*-acetyl migration. In bacteria, *N*-acetyl-serine then acts as an inducer of transcription of assimilatory sulfate reduction genes (Kredich 1996). In accordance, relative contents of *O*-acetyl-serine as well as *N*-acetyl-serine were drastically reduced during growth on sulfide, thiosulfate and elemental sulfur resulting in shut down of the sulfate reduction pathway (Figs. 1b, 4). In plants *O*-acteyl-serine acts as a regulator for assimilatory sulfate reduction (Hubberten et al. 2012; Kopriva, 2006).

In contrast to the situation in *E. coli* and many other bacteria, where a transsulfuration pathway via cystathionine exists (Hwang et al. 2002; Manders et al. 2013), biosyntheses of methionine and cysteine are not immediately intertwined in *A. vinosum* (Fig. 1b, c). In this organism, the formation of homocysteine by the enzyme *O*-succinyl-l-homoserine sulfhydrylase (MetZ, Alvin_1027) appears to be the only entry point for incorporation of sulfide into methionine (Fig. 1c). Homocysteine then serves as the immediate precursor for methionine by accepting a methyl group from N5-methyl-5,6,7,8-tetrahydrofolate catalyzed by either cobalamin-dependent (MetH: Alvin_1622) or cobalamin-independent (MetE: Alvin_2262) methionine synthase (Pejchal and Ludwig 2005).

Homocysteine is the most abundant amino acid in *A. vinosum* (up to five times more abundant than the proteinogenic glutamic acid and aspartic acid, Table S1). Metabolite fluxes directed to the formation of homocysteine appeared quite stable under the different growth conditions studied (Fig. 1c). Methionine and homocysteine are both very important intermediates in methyl transfer reactions involving *S*-adenosylmethionine (AdoMet) as the methyl group donor (Fig. 1c). These transfer reactions have long been known to play an especially important role in anoxygenic phototrophic bacteria like *A. vinosum* because methyl transfer to magnesium protoporphyrin IX yielding Mg protoporphyrin IX 13-methylester (catalyzed by BchM, Alvin_2638) is the first step specific for bacteriochlorophyll synthesis (Sganga et al. 1992). AdoMet is transformed into *S*-adenosylhomocysteine (AdoHomoCys) in the course of this reaction. AdoHomoCys non-competitively inhibits methyl transfer (Sganga et al. 1992) and is immediately hydrolytically recycled to homocysteine (catalyzed by AhcY, Alvin_0320). Furthermore, high concentrations of AdoMet are known to inhibit threonine biosynthesis in *A. vinosum* by negatively influencing homoserine dehydrogenase activity (Sugimoto et al. 1976). Taken together, the high demand of bacteriochlorophyll as well as the inhibitory effects of AdoMet and AdoHomoCys may serve as explanations for the high intracellular levels of homocysteine in the phototroph *A. vinosum*.

#### Glutathione

Glutathione and its precursor gamma-glutamylcysteine are of special interest in *A. vinosum*, because glutathione in its persulfidic form has been speculated to be involved in transport of sulfane sulfur across the cytoplasmic membrane in purple sulfur bacteria (Frigaard and Dahl 2009). Glutathione is synthesized in two reaction steps requiring cysteine, glutamine, glycine and the enzymes glutamate/cysteine ligase and glutathione synthetase encoded by Alvin_0800 and Alvin_0197, respectively (Fig 1b). Glutathione disulfide could be formed via the action of glutathione peroxidase (Alvin_2032) or thiol peroxidase (Gar A, Alvin_1324) and could be reduced back to glutathione by glutathione-disulfide reductase (GarB, Alvin_1323) (Chung and Hurlbert 1975; Vergauwen et al. 2001). However, γ-glutamylcysteine and glutathione concentrations were similar under all growth conditions not yielding further support for a major role of glutathione in oxidative sulfur metabolism (Figs. 1b, 4b). In contrast to a previous report, we were not able to detect any glutathione amide in *A. vinosum* (Bartsch et al. 1996). Besides the identified sulfur-containing metabolites, we also detected an unknown thiol (UN) that predominated during growth on sulfide (Fig. 4b). Since this metabolite was also detected in similar concentrations in wild type cells on malate (Fig. 4b), a specific role in the oxidation of sulfide cannot be concluded.

#### Central carbon metabolism

**Fig. 5:**
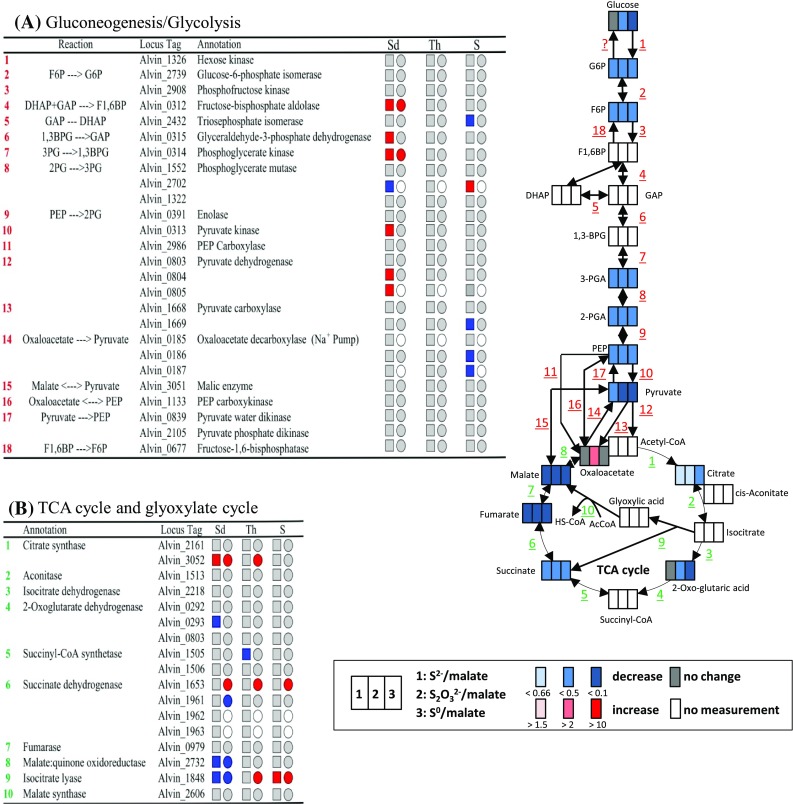
Comparison between metabolite, transcript (Weissgerber et al. 2013) and protein (Weissgerber et al. 2014) data of glycolysis/gluconeogenesis (**a**) and the citric acid cycle/glyoxylic acid cycles (**b**). Reactions of gluconeogenesis are additionally outlined in table (**a**). The transcriptomic (*boxes*) (Weissgerber et al. 2013) and proteomic (*circles*) (Weissgerber et al. 2014) profiles (all relative to growth on malate) are depicted next to the respective locus tag. Relative fold changes in mRNA levels above 2 (*red*) were considered significantly enhanced. Relative changes smaller than 0.5 (*blue*) were considered as indicating significant decreases in mRNA levels. Relative fold changes between 0.5 and 2 (*grey*) indicated unchanged mRNA levels. The same color coding is applied to changes on the protein levels. Here, values above 1.5 (*red*) and below 0.67 (*blue*) were considered significant. Those cases, where transcriptomic data was not available or the respective protein not detected in the proteomic approach, respectively, are indicated by *white squares* or *circles*. *Sd* sulfide, *Th* thiosulfate, *S* elemental sulfur

With regard to central carbon metabolism the relative amount of all detected intermediates of gluconeogenesis/glycolysis and the citric acid cycle decreased at least twofold during photolithoautotrophic growth on reduced sulfur compounds (Fig. 5). Oxalic acid, citric acid and 2-oxo-glutaric acid were the only exceptions to this rule. When present as an external substrate, malate enters central carbon metabolism via the formation of pyruvate catalyzed by the NADP-dependent malic enzyme (Sahl and Trüper 1980). However, the relative mRNA and protein levels for this enzyme were not affected by the switch from heterotrophic growth on malate to autotrophic growth on carbon dioxide (Fig. 5a) indicating that it also exerts an important, if not essential role, in the absence of external malate (Weissgerber et al. 2013, 2014). The reaction has a standard free-energy change of about −8 kJ mol^−1^ in the decarboxylation direction (Kunkee 1967). When compared to growth on malate, the ratio of pyruvic acid over malic acid in *A. vinosum* changes from about 1–100 during growth on sulfur compounds (Table S1). If we assume similar CO_2_, NADP^+^ and NADPH concentrations under malate and sulfur-oxidizing conditions, the ∆G value would become positive (according to ∆G = −8 kJ mol^−1^ + 2.303 *RT* log(100) = +3.38 kJ mol^−1^), thus favoring the reverse carboxylating reaction. We therefore propose that under autotrophic conditions malic enzyme catalyzes the NADPH_2_-dependent reductive carboxylation of pyruvate to malate, as has been reported for engineered *Saccharomyces cerevisiae* strains (Zelle et al. 2011) and also for *Roseobacter denitrificans*. The latter organism uses anaplerotic pathways mainly via malic enzyme to fix 10–15 % of protein carbon from CO_2_ (Tang et al. 2009). In addition to PEP-carboxylase, PEP-carboxykinase and pyruvate carboxylase (Tang et al. 2011), malic enzyme also appears to be a major player during anaplerotic carbon dioxide fixation in *A. vinosum* (Fig. 5). Formation of malate by the malic enzyme represents the most efficient anaplerotic reaction for replenishing the citric acid cycle with oxaloacetate, because the reaction does not consume ATP.

The glyoxylate cycle is a further pathway suited for replenishing the TCA cycle, when central intermediates of this pathway are needed as building blocks for anaplerotic reactions. Indeed, the presence of isocitrate lyase and malate synthase in *A. vinosum* proves an active glyoxylate cycle, just as has been reported for several purple non-sulfur bacteria, e.g. *Rhodopseudomonas palustris* (McKinlay and Harwood 2011). Notably, relative transcript and protein levels for isocitrate lyase (Alvin_1848), the key enzyme of the glyoxylate cycle in *A. vinosum* (Fuller et al. 1961), significantly increased in the presence of elemental sulfur, while levels decreased on sulfide (Fig. 5b). Isocitrate lyase is long known to be adaptively formed under conditions necessitating net synthesis of C4 compounds (Kornberg 1959). The glyoxylate cycle as a whole has a bypass function that prevents loss of carbon dioxide and production of NAD[P]H_2_ otherwise occurring through the isocitrate dehydrogenase and 2-oxoglutarate dehydrogenase catalyzed reactions. This bypass function appears to be especially important during growth on elemental sulfur, while the cells appear to shut down this possibility in the presence of sulfide. In anoxygenic anaerobic phototrophs, like *A. vinosum*, photosynthesis generates reducing equivalents through light-induced electron transport. Channeling of these reducing equivalents into autotrophic CO_2_ fixation is very important, because respiration is not possible. Elemental sulfur is not as a potent reductant as sulfide and thus, consuming excess reducing equivalents produced by photosynthesis is less essential on elemental sulfur. We propose, that the gate into the glyoxylate cycle is narrowed in the presence of sulfide resulting in loss of already fixed carbon through the TCA cycle and thereby enabling the cells to shuffle more (excess) reducing equivalents to CO_2_. A strategy similar in principle has been reported for *R. palustris*, where the Calvin–Benson cycle is not only assimilating CO_2_ and producing biomass during photoautotrophic growth, but is also accepting reducing equivalents during photoheterotrophic growth (McKinlay and Harwood 2010). In accordance, the relative amount of 2-oxo-glutaric acid remained unchanged on sulfide and decreased on thiosulfate and elemental sulfur (Fig. 2).

#### Gluconeogenesis

In the course of gluconeogenesis, phosphoenolpyruvate formation is catalyzed by pyruvate water dikinase (Alvin_0839) or pyruvate, phosphate dikinase (Alvin_2105) in *A. vinosum* (Fig. 5). In our transcriptome and proteome studies, we noted a decreasing tendency of relative mRNA and protein levels for pyruvate water dikinase during growth on reduced sulfur compounds, although values did not reach thresholds (Weissgerber et al. 2013, 2014). Down-regulation of the transcript and protein levels of the pyruvate water dikinase appears to be a consequence of low intracellular malic acid concentrations. Along this line, relative amounts of pyruvic acid and phosphoenolpyruvic acid were also significantly lower on reduced sulfur compounds than on malate (Fig. 5; Fig. S1; Table S1). Relative abundance for 2- and 3-phosphoglyceric acid corresponded to that of phosphoenolpyruvic acid (Fig. 5; Fig. S1; Table S1). In accordance, drastic changes of mRNA and protein levels for enolase (Alvin_0391), and phosphoglycerate mutases (Alvin_1322, Alvin_2702, Alvin_1552) were not detected (Fig. 5a) (Weissgerber et al. 2013, 2014). The ratios between relative amounts of fructose-6-phosphate and glucose-6-phosphate were similar even under the different growth conditions. The relative amounts of the hexose-phosphates were significantly lower during autotrophic growth and thus, followed the same pattern as the earlier intermediates of gluconeogenesis, e.g., 2- and 3-phospholgycerate (Fig. 5; Fig. S1; Table S1). We also found low relative intracellular amounts of glucose and fructose under all growth conditions (Table S1). Detection of glucose in the cells supports the hypothesis, that the known incapability of *A. vinosum* to grow on externally available glucose (Imhoff 2005) is due to the lack of a phosphotransferase system for glucose uptake (Weissgerber et al. 2011). In contrast to the phosphorylated hexoses, almost similar relative intracellular glucose, mannose, and fructose abundances were measured for the *A. vinosum* wild type on malate and sulfide, while relative amounts were significantly lower in cells grown on elemental sulfur or thiosulfate (Fig. 2; Fig. S2; Table S1).

#### Free amino acids

Upon the switch from photoorganoheterotrophic to photolithoautotrophic growth, we observed a drastic decrease (20–39 %) in the total concentration of free standard amino acids. An especially drastic decrease was observed for all amino acids of the pyruvic acid family, corresponding to the low relative pyruvic acid content in sulfur-grown cells (Fig. 2; Figs. S1, S2; Table S1). This may indicate a major drainage of malate into pyruvate and the respective down-stream amino acids. Leucine and its precursors 2-oxoisocaproate and 2-isopropylmalate showed basically parallel changes of relative content, i.e. all three compounds were less abundant in autotrophically grown cells (Table S1). This is in complete accordance with an earlier work (Stieglitz and Calvo 1974), that reported suppression of 2-oxoisovalerate transformation to 2-isopropylmalate, the first step of leucine biosynthesis, by leucine in *A. vinosum*. Among the amino acids derived from oxalic acid, aspartic acid exhibited a diminished concentration in cells grown on reduced sulfur compounds (Table S1). This may be explained by downregulation of the aminotransferase catalyzing the formation of aspartate from oxalic acid (Alvin_0361): the relative mRNA and protein levels for the corresponding gene/protein were lower during growth on sulfur compounds than in the presence of malate (Weissgerber et al. 2013, 2014). While relative amounts of 2-oxo-glutaric acid and its derivatives glutamate and arginine were quite similar for the different growth conditions, the ATP consuming synthesis of the product glutamine predominated in cells cultivated on malate (Fig. 3; Fig. S1; Table S1). 4-Aminobutyric acid was detected both on malate and sulfide (Table S1). This compound is usually formed by decarboxylation of glutamate (Dhakal et al. 2012), but we have not yet been able to identify the corresponding enzyme/gene in *A. vinosum*. Concentrations of serine, the first intermediate of the 3-phosphoglyceric acid amino acid family, were also lower under autotrophic than under heterotrophic conditions and paralleled the changes noticed for the precursor 3-phosphoglyceric acid (Table S1). In line with this observation, relative mRNA and protein levels for the enzymes involved in these reaction steps (Alvin_2085/_1956/_1986/_2518) were unchanged (Weissgerber et al. 2013, 2014). Concentrations of aromatic amino acids requiring phosphoenolpyruvic acid as a precursor were similar on malate and on the different reduced sulfur compounds (Fig. 2; Fig. S1; Table S1). The same holds true for the ribose-5-phosphate derivative histidine (Fig. 2; Fig. S1; Table S1).

#### Fatty acids

Transport of hydrophobic compounds such as elemental sulfur may require changes of outer and/or inner membrane fatty acid composition (Frigaard and Dahl 2009). However, with the only exception of an increased relative amount for hexanoic acid after growth on thiosulfate, the relative contents of the various detected fatty acids were quite similar under all conditions (Fig. 2; Fig. S1; Table S1). The same holds true for glycerol and glycerol-3-phosphate, precursors for phosphoglycerolipids and also for ethanolamine, a component of the latter (Fig 2; Fig. S1; Table S1). Thus, composition of lipids in both membranes appears to remain unaltered regardless of whether *A. vinosum* is cultivated photoorganoheterotrophically on malate or photolithoautotrophically on sulfur compounds.

Notably, relative abundance of three further unidentified metabolites (A142003-101, A145008-101, A255002-101), oxalic acid, xylose, uracil and phosphate specifically increased after growth on thiosulfate, while their relative amount remained unaffected or decreased in the presence of sulfide or elemental sulfur compared to growth on malate (Fig. 2; Fig. S3). Currently, we have no explanation for this effect.

### Comparison of metabolites of the wild type after growth on different sulfur compounds

When scoring differences of metabolite amounts observed in cells grown on different sulfur compounds (Figs. S4, S5), the most prominent observation was, that cells grown on elemental sulfur exhibited a much lower energy level than cells grown on sulfide or thiosulfate. More specifically, intracellular relative amounts of the high energy compounds citric acid and pyrophosphate were very low on elemental sulfur. Usually, *A. vinosum* keeps an energy charge (([ATP] + 0.5 [ADP])/([ATP] + [ADP] + [AMP])) of 0.9 during growth on malate or thiosulfate in the light (Gibson and Morita 1967). Absolute ATP concentrations in the range of 8 and 10 nmol mg^−1^ protein were reported for *A. vinosum* strains DSM 185 and DSM 180 grown in the light on sulfide or on a sulfide/succinate/pyruvate medium, respectively (Miović and Gibson 1971; van Gemerden 1980). ADP concentrations were found to be in a range of 2–4 nmol mg^−1^ protein on sulfide/succinate/pyruvate, thiosulfate as well as on malate (Gibson and Morita 1967; Miović and Gibson 1971). In the light, AMP concentrations were lower than ADP concentrations on all of these substrates. In accordance, AMP was not detected in sulfide, thiosulfate and malate grown wild type cells in the present study indicating a high cellular energy charge on these substrates. In contrast, AMP was readily detected on elemental sulfur further supporting a low energy level of the cells on this substrate. In addition, the intracellular relative contents of sugars (e.g. glucose, fructose, ribose, mannose), polyhydroxy acids and free amino acids were significantly lower in elemental sulfur-grown than in sulfide- or thiosulfate-grown cells (Fig. S1; Table S1). While electrons stemming from sulfide oxidation are fed immediately into the quinone pool via the sulfide:quinone oxidoreductase catalyzed reaction (Fig. 1a) (Frigaard and Dahl 2009), electrons derived from thiosulfate are channeled to more electropositive *c*-cytochromes via the Sox system or TsdA (Fig. 1a) (Denkmann et al. 2012; Hensen et al. 2006; Welte et al. 2009). In case of elemental sulfur, it is highly probable that uptake into the cell requires input of energy before its oxidation can start. Experiments with the uncoupler carbonyl cyanide 3-chlorophenylhydrazone (CCCP) resulted in an inability of *Acidithiobacillus caldus* to oxidize elemental sulfur (Hallberg et al. 1996). Preliminary experiments with *A. vinosum* indicated a similar effect on metabolism of elemental sulfur, but no impact of CCCP on the oxidation of sulfide and thiosulfate (Bettina Franz and Christiane Dahl, Institute for Micorbiology & Biotechnology, University of Bonn, unpublished). Thus, energy-requiring biosyntheses can most efficiently be performed in the presence of sulfide, followed by thiosulfate and finally elemental sulfur as oxidizable substrates. This conclusion is corroborated by our previous finding that compared to growth on malate, sulfide but not elemental sulfur led to increased relative mRNA and protein levels for the genes/proteins participating in the gluconeogenetic conversion of 3-phosphogylceric acid to fructose-1,6-bisphosphate (Alvin_0314/_0315/_0312) (Fig. 5a) (Weissgerber et al. 2013, 2014).

It may at first appear surprising that the highest amount of intracellular hydrogen sulfide was detected for the wild type growing on thiosulfate (Fig. 4b). However, it should be kept in mind that cultures initially supplemented with sulfide had already used up external sulfide and were oxidizing intracellular sulfur reserves at the time point of sampling. Based on the current model thiosulfate is oxidized via the Sox system (Fig. 1a) (Hensen et al. 2006; Welte et al. 2009), hence there is currently no good explanation for formation of sulfide during thiosulfate oxidation. In accordance with the presence of free intracellular hydrogen sulfide, and the possible incorporation of sulfane sulfur stemming from thiosulfate into cysteine via the formation of *S*-sulfocysteine, the concentration of cysteine was also highest on thiosulfate (Figs. 1b, 4b; Fig. S1; Table S1). Notably, unidentified metabolite A166004-101 was very abundant on sulfide, while unidentified metabolite A277004-101 predominated on thiosulfate and elemental sulfur (Fig. S3; Table S1).

### Comparison of wild type and Δ*dsrJ* mutant after growth on sulfide

**Fig. 6:**
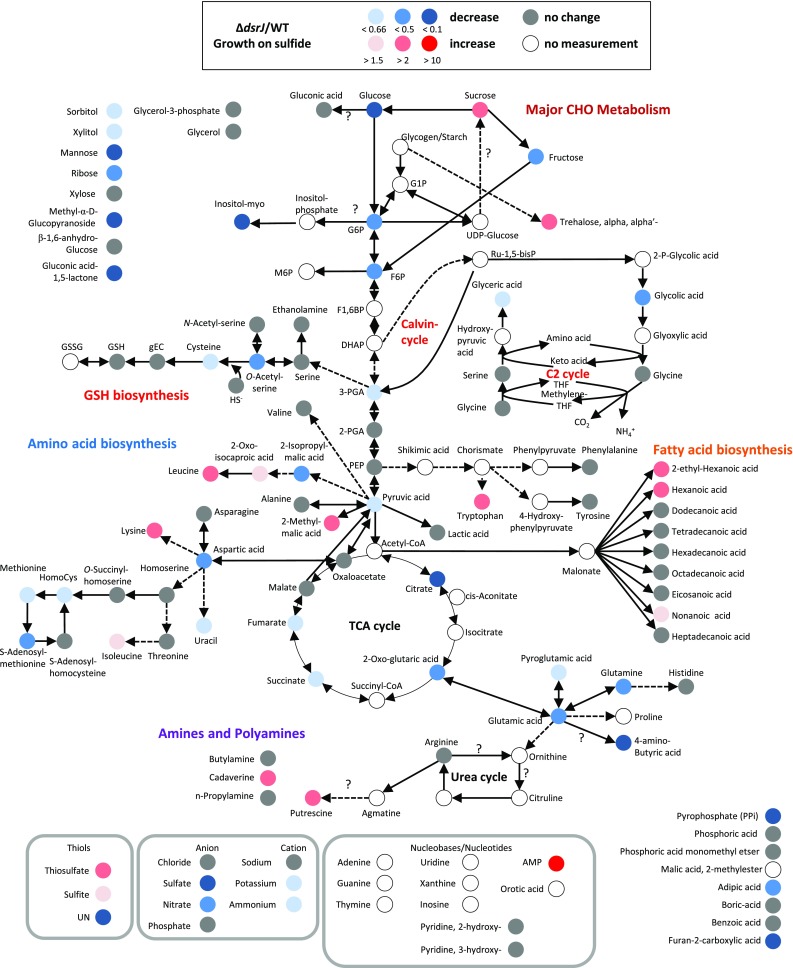
Simplified scheme of *A. vinosum* central metabolism comparing metabolite concentrations after growth on sulfide for the Δ*dsrJ* mutant strain with those for the wild type. Color range visualizes changes of at least 1.5-fold, twofold and tenfold, respectively

As the final step, we evaluated the metabolomic patterns of the sulfur oxidation deficient *A. vinosum* Δ*dsrJ* strain during growth on sulfide. When including the metabolite data of the *dsrJ* mutant into a PCA analysis (Fig. 3d), the score plot is slightly altered compared to Fig. 3c as the calculation is dependent on the whole data provided. Still the distribution of the wild type *A. vinosum* under different conditions resembles that of Fig. 3c. Interestingly the metabolome of the *dsrJ* mutant can hardly be separated from *A. vinosum* grown on elemental sulfur, though the experimental variation is lower, again indicating that elemental sulfur is a difficult substrate. Probably, the *dsrJ* mutant prevents or slows down regeneration of the sulfane sulfur acceptor DsrC (Fig. 1), while provision of bioavailable reduced sulfur from elemental sulfur seems to be similarly reduced due to the inertness of the substrate requiring additional energy to make use of it. These global changes are further visualized in Fig. 6. The following general observations were noted: Due to the complete inability of the Δ*dsrJ* mutant to further metabolize stored sulfur (Sander et al., 2006), concentrations of all the downstream oxidized sulfur compounds (sulfite and sulfate) were diminished. As a consequence, mutant cells had to cope with a low intracellular energy state, which correlates to some extent with a wild type growing on elemental sulfur, reflected both by pyrophosphate and citric acid levels below detection limits and a high AMP level (Fig. 6; Fig. S1; Table S1). The lack of energy in the mutant strain is furthermore clearly illustrated by reduced relative amounts of metabolites requiring energy-consuming steps for their biosynthesis. For example, content of sugars is reduced to only 35 % and that of free amino acids to only 59 % of that of the wild type (Fig. S2; Table S1). Relative amounts of most gluconeogenic intermediates were also diminished. As an example, the Δ*dsrJ* mutant grown on sulfide contained the lowest relative contents found for fructose-6-phosphate and glucose-6-phosphate (Figs. S1; Table S1). All the more surprising, we detected elevated intracellular leucine, lysine and tryptophane concentrations for the mutant on sulfide (Fig. 6). Interestingly, levels of two osmotically active compounds (sucrose and trehalose) were enhanced for the mutant, which can be taken as indirect evidence for low ion concentrations in the cells that are counteracted by accumulation of organic solutes. Indeed, the sum of the concentrations of potassium, ammonium, nitrate and sulfate was significantly lower in the mutant strain than in wild type *A. vinosum* (Fig. 2; Fig. S2; Table S1).

## Concluding remarks

Metabolic profiles obtained for the purple sulfur bacterium *A. vinosum* upon exposure to malate, sulfide, thiosulfate, elemental sulfur and for a Δ*dsrJ* mutant upon sulfide provided global insights into metabolite changes triggered by alteration of electron donors and carbon source. The data generated during this study confirmed changes expected for sulfate and cysteine concentrations upon a switch from photoorganoheterotrophic growth on malate and sulfate to photolithoautotrophic growth in the presence of reduced sulfur compounds. Furthermore, this work provided first insights into the general availability and ratio of different metabolites in *A. vinosum* comprising intermediates of the citric acid and glyoxylate cycles, gluconeogenesis as well as amino acid and fatty acid biosyntheses. A clear correlation was observed between the energy level of the electron donor provided and the intracellular relative contents of amino acid and sugars. In higher organisms, such as plants, the transition between transcriptional changes, proteomic changes and finally alterations of the metabolite compositions is less straight forward (Fernie and Stitt 2012) and rather maintenance of homeostasis is pursued (Hoefgen and Nikiforova 2008). In *A. vinosum*, though, we found a more continuous correlation between changes at the transcriptome and proteome levels and metabolic adjustments in response to environmental conditions.

## Electronic supplementary material

Below is the link to the electronic supplementary material.

Supplementary material 1 (PPTX 409 kb)

Supplementary material 2 (XLS 1695 kb)
