# Supplementary material for: Metabolomic profiling of the purple sulfur bacterium Allochromatium vinosum during growth on different reduced sulfur compounds and malate
Source: Metabolomics. 2014 May 22;10(6):1094–112. doi: 10.1007/s11306-014-0649-7 (PMC4213376; doi:10.1007/s11306-014-0649-7)
Supplement: Supplementary file 1 — Supplementary material 1 (PPTX 409 kb) [file 11306_2014_649_MOESM1_ESM.pptx]

## Slide 1
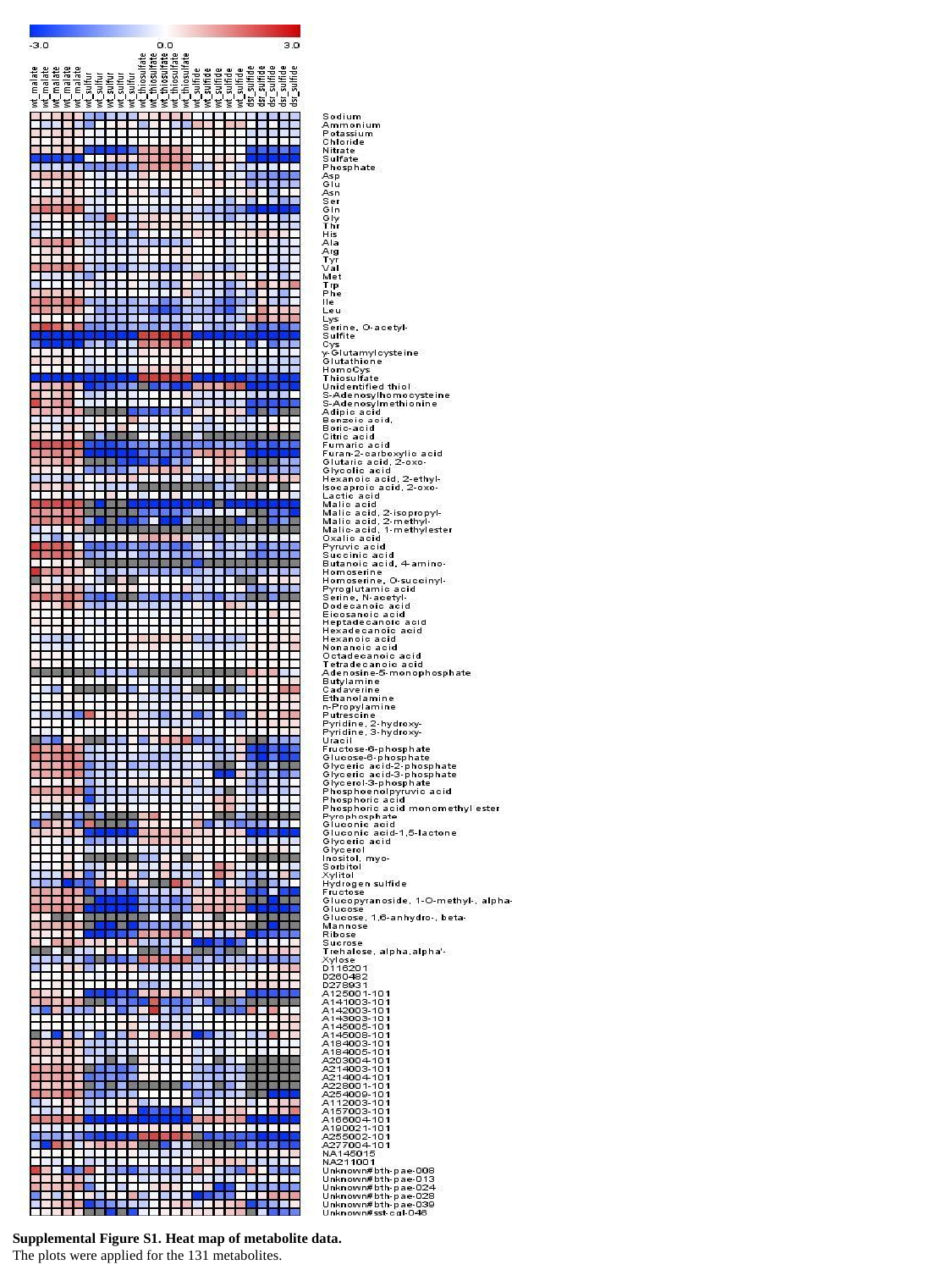

Supplemental Figure S1. Heat map of metabolite data.
The plots were applied for the 131 metabolites.

## Slide 2
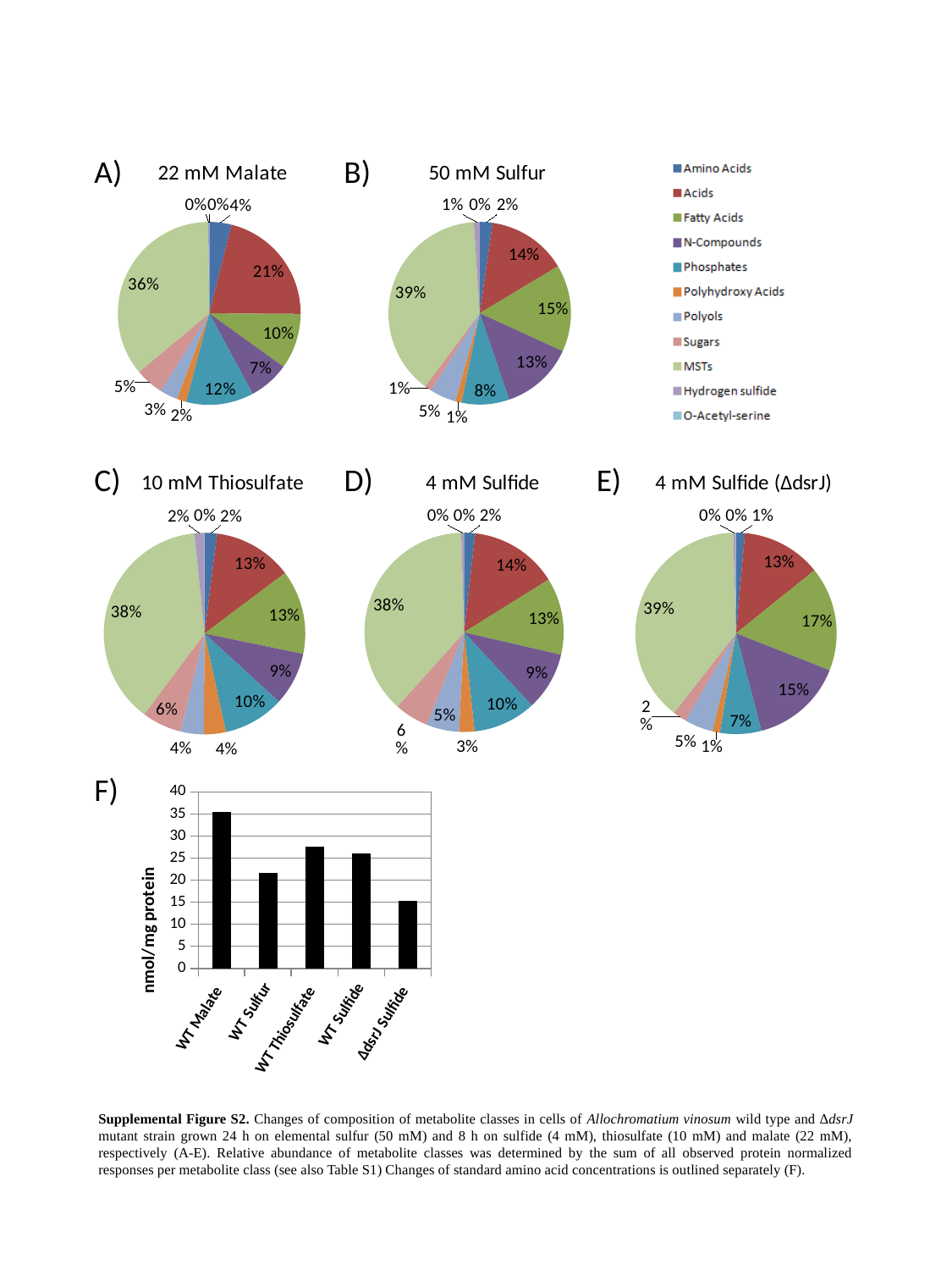

A)
### Chart: 22 mM Malate
| Category | |
|---|---|
| Amino Acids | 22.348260596555686 |
| Acids | 120.08014461608433 |
| Fatty Acids | 55.59054092932354 |
| N-Compounds | 40.827119533461435 |
| Phosphates | 67.8480659206414 |
| Polyhydroxy Acids | 9.60666988617373 |
| Polyols | 17.814561706996294 |
| Sugars | 28.572721842417486 |
| MSTs | 203.47341180150835 |
| Hydrogen sulfide | 0.932566600112155 |
| O-Acetyl-serine | 0.2028694227227809 |B)
### Chart: 50 mM Sulfur
| Category | |
|---|---|
| Amino Acids | 7.281079571945785 |
| Acids | 44.96764762959405 |
| Fatty Acids | 49.21382699796279 |
| N-Compounds | 41.209737757704644 |
| Phosphates | 26.91377766715609 |
| Polyhydroxy Acids | 3.5305671071393405 |
| Polyols | 14.712614208810496 |
| Sugars | 4.044271134982593 |
| MSTs | 123.54000425270858 |
| Hydrogen sulfide | 3.294642578927981 |
| O-Acetyl-serine | 0.02558736259349685 |
C)
D)
E)
### Chart: 10 mM Thiosulfate
| Category | |
|---|---|
| Amino Acids | 7.7028078452656485 |
| Acids | 49.02314492865521 |
| Fatty Acids | 51.8909748778747 |
| N-Compounds | 32.857236869588874 |
| Phosphates | 37.737940283324654 |
| Polyhydroxy Acids | 13.582362355874825 |
| Polyols | 14.300189718481302 |
| Sugars | 24.511894273104645 |
| MSTs | 146.86156156609218 |
| Hydrogen sulfide | 6.149326934866616 |
| O-Acetyl-serine | 0.027393653597220242 |
### Chart: 4 mM Sulfide
| Category | |
|---|---|
| Amino Acids | 6.8910285765737616 |
| Acids | 54.87702239364272 |
| Fatty Acids | 47.89904597768772 |
| N-Compounds | 36.32291003111001 |
| Phosphates | 39.07855426533549 |
| Polyhydroxy Acids | 9.634788141431292 |
| Polyols | 20.27255422046275 |
| Sugars | 21.261806615917717 |
| MSTs | 145.14118399551958 |
| Hydrogen sulfide | 1.7196078930046494 |
| O-Acetyl-serine | 0.03985339916849044 |
### Chart: 4 mM Sulfide (ΔdsrJ)
| Category | |
|---|---|
| Acids | 4.8898851241457555 |
| Fatty Acids | 43.70203373006104 |
| N-Compounds | 57.22733751030035 |
| Phosphates | 51.154464997691875 |
| Polyhydroxy Acids | 22.62424909038876 |
| Polyols | 4.1008652022961085 |
| Sugars | 15.749573551562568 |
| MSTs | 7.416216044647729 |
| Hydrogen sulfide | 133.2513751143095 |
| O-Acetyl-serine | 1.5585558557649257 |F)
### Chart
| Category | |
|---|---|
| WT Malate | 35.40339324229986 |
| WT Sulfur | 21.694824894262144 |
| WT Thiosulfate | 27.689248293512726 |
| WT Sulfide | 26.046431395676876 |
| ΔdsrJ Sulfide | 15.311839706222505 |Supplemental Figure S2. Changes of composition of metabolite classes in cells of Allochromatium vinosum wild type and ΔdsrJ mutant strain grown 24 h on elemental sulfur (50 mM) and 8 h on sulfide (4 mM), thiosulfate (10 mM) and malate (22 mM), respectively (A-E). Relative abundance of metabolite classes was determined by the sum of all observed protein normalized responses per metabolite class (see also Table S1) Changes of standard amino acid concentrations is outlined separately (F).

## Slide 3
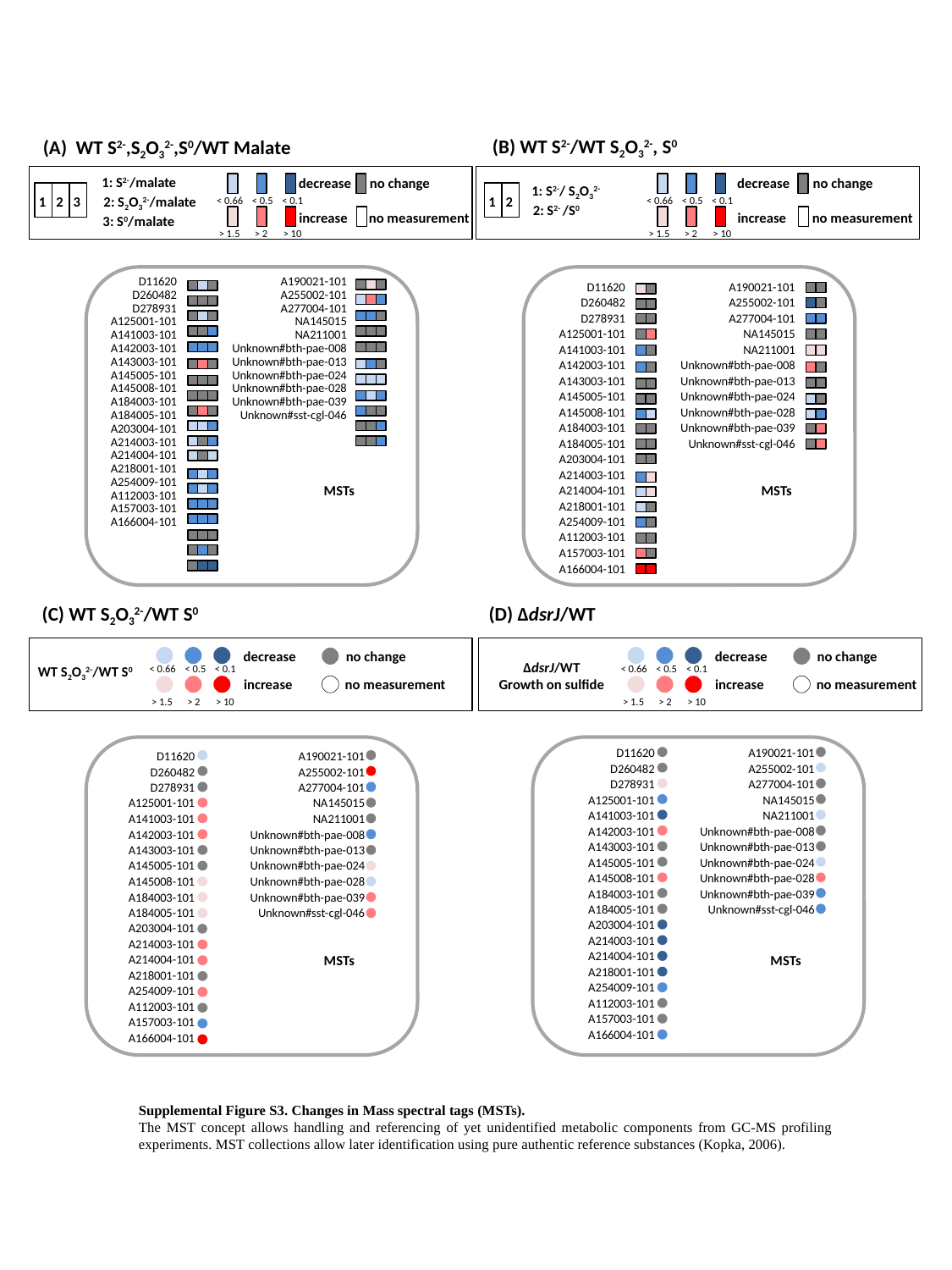

(B) WT S2-/WT S2O32-, S0
(A) WT S2-,S2O32-,S0/WT Malate
1: S2-/malate
decrease
no change
1
2
3
2: S2O32-/malate
< 0.66
< 0.5
< 0.1
increase
no measurement
3: S0/malate
> 1.5
> 2
> 10
decrease
no change
1: S2-/ S2O32-
1
2
< 0.66
< 0.5
< 0.1
2: S2- /S0
increase
no measurement
> 1.5
> 2
> 10
MSTs
MSTs
| D11620 |
| --- |
| D260482 |
| D278931 |
| A125001-101 |
| A141003-101 |
| A142003-101 |
| A143003-101 |
| A145005-101 |
| A145008-101 |
| A184003-101 |
| A184005-101 |
| A203004-101 |
| A214003-101 |
| A214004-101 |
| A218001-101 |
| A254009-101 |
| A112003-101 |
| A157003-101 |
| A166004-101 |
| A190021-101 |
| --- |
| A255002-101 |
| A277004-101 |
| NA145015 |
| NA211001 |
| Unknown#bth-pae-008 |
| Unknown#bth-pae-013 |
| Unknown#bth-pae-024 |
| Unknown#bth-pae-028 |
| Unknown#bth-pae-039 |
| Unknown#sst-cgl-046 |
| |
| |
| |
| |
| |
| D11620 |
| --- |
| D260482 |
| D278931 |
| A125001-101 |
| A141003-101 |
| A142003-101 |
| A143003-101 |
| A145005-101 |
| A145008-101 |
| A184003-101 |
| A184005-101 |
| A203004-101 |
| A214003-101 |
| A214004-101 |
| A218001-101 |
| A254009-101 |
| A112003-101 |
| A157003-101 |
| A166004-101 |
| A190021-101 |
| --- |
| A255002-101 |
| A277004-101 |
| NA145015 |
| NA211001 |
| Unknown#bth-pae-008 |
| Unknown#bth-pae-013 |
| Unknown#bth-pae-024 |
| Unknown#bth-pae-028 |
| Unknown#bth-pae-039 |
| Unknown#sst-cgl-046 |
| |
| |
| |
| |
| |
(C) WT S2O32-/WT S0
(D) ΔdsrJ/WT
decrease
no change
< 0.66
< 0.5
< 0.1
WT S2O32-/WT S0
increase
no measurement
> 1.5
> 2
> 10
decrease
no change
ΔdsrJ/WT
Growth on sulfide
< 0.66
< 0.5
< 0.1
increase
no measurement
> 1.5
> 2
> 10
MSTs
MSTs
| D11620 |
| --- |
| D260482 |
| D278931 |
| A125001-101 |
| A141003-101 |
| A142003-101 |
| A143003-101 |
| A145005-101 |
| A145008-101 |
| A184003-101 |
| A184005-101 |
| A203004-101 |
| A214003-101 |
| A214004-101 |
| A218001-101 |
| A254009-101 |
| A112003-101 |
| A157003-101 |
| A166004-101 |
| A190021-101 |
| --- |
| A255002-101 |
| A277004-101 |
| NA145015 |
| NA211001 |
| Unknown#bth-pae-008 |
| Unknown#bth-pae-013 |
| Unknown#bth-pae-024 |
| Unknown#bth-pae-028 |
| Unknown#bth-pae-039 |
| Unknown#sst-cgl-046 |
| |
| |
| |
| |
| |
| D11620 |
| --- |
| D260482 |
| D278931 |
| A125001-101 |
| A141003-101 |
| A142003-101 |
| A143003-101 |
| A145005-101 |
| A145008-101 |
| A184003-101 |
| A184005-101 |
| A203004-101 |
| A214003-101 |
| A214004-101 |
| A218001-101 |
| A254009-101 |
| A112003-101 |
| A157003-101 |
| A166004-101 |
| A190021-101 |
| --- |
| A255002-101 |
| A277004-101 |
| NA145015 |
| NA211001 |
| Unknown#bth-pae-008 |
| Unknown#bth-pae-013 |
| Unknown#bth-pae-024 |
| Unknown#bth-pae-028 |
| Unknown#bth-pae-039 |
| Unknown#sst-cgl-046 |
| |
| |
| |
| |
| |
Supplemental Figure S3. Changes in Mass spectral tags (MSTs).
The MST concept allows handling and referencing of yet unidentified metabolic components from GC-MS profiling experiments. MST collections allow later identification using pure authentic reference substances (Kopka, 2006).

## Slide 4
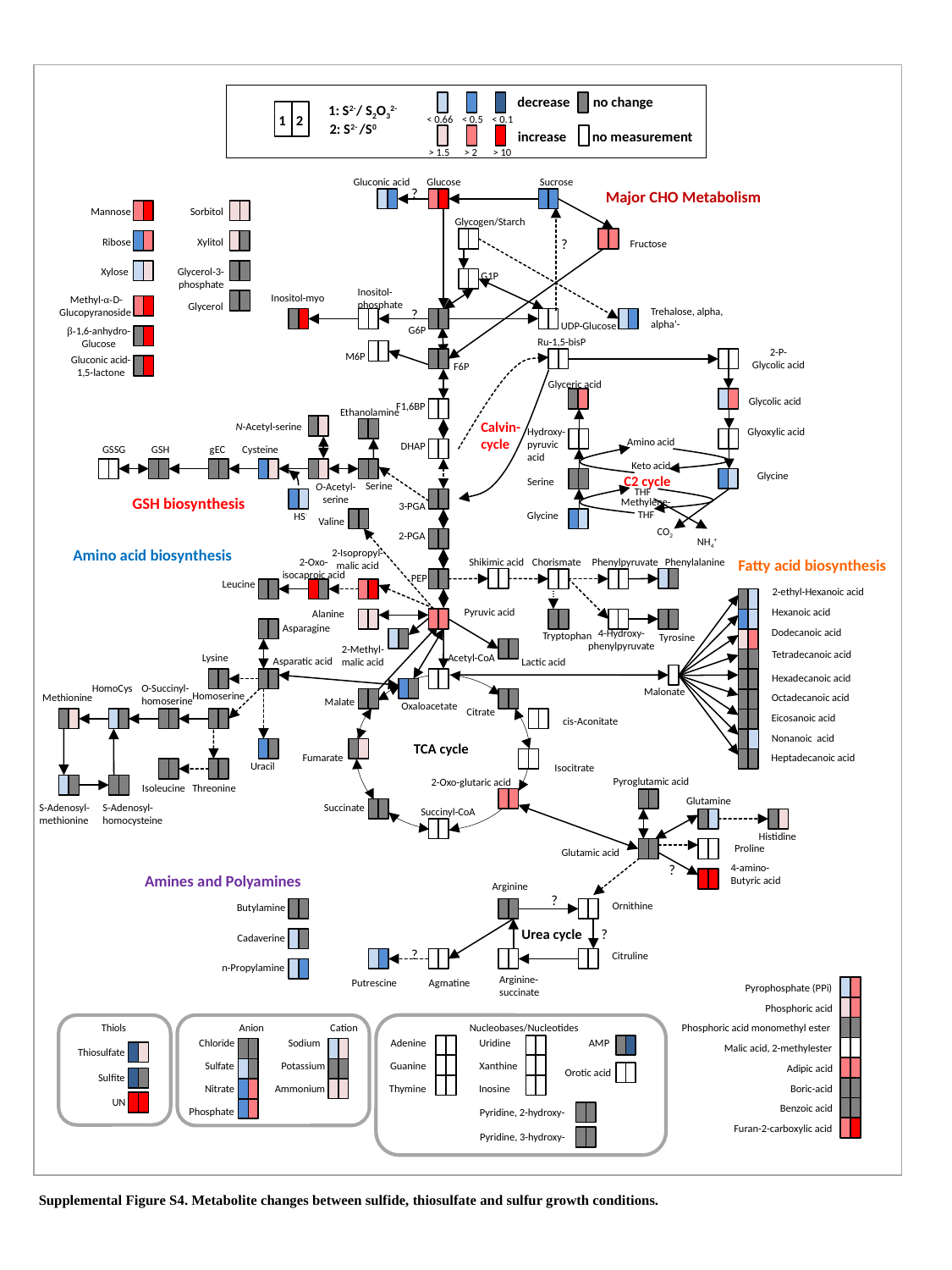

decrease
no change
1: S2-/ S2O32-
1
2
< 0.66
< 0.5
< 0.1
2: S2- /S0
increase
no measurement
> 1.5
> 2
> 10
Gluconic acid
Glucose
Sucrose
?
Major CHO Metabolism
Mannose
Sorbitol
Ribose
Xylitol
Xylose
Glycerol-3-phosphate
Methyl-α-D-
Glucopyranoside
Glycerol
β-1,6-anhydro-
Glucose
Gluconic acid-
1,5-lactone
Glycogen/Starch
?
Fructose
G1P
Inositol-phosphate
Inositol-myo
Trehalose, alpha, alpha’-
?
UDP-Glucose
G6P
Ru-1,5-bisP
2-P-
Glycolic acid
M6P
F6P
Glyceric acid
Glycolic acid
F1,6BP
Ethanolamine
Calvin-
cycle
N-Acetyl-serine
Hydroxy-
pyruvic
acid
Glyoxylic acid
Amino acid
DHAP
GSSG
GSH
gEC
Cysteine
Keto acid
Glycine
C2 cycle
Serine
Serine
O-Acetyl-serine
THF
GSH biosynthesis
Methylene-
THF
3-PGA
Glycine
HS-
Valine
CO2
2-PGA
NH4+
Amino acid biosynthesis
2-Isopropyl-
malic acid
2-Oxo-
isocaproic acid
Shikimic acid
Chorismate
Phenylpyruvate
Phenylalanine
Fatty acid biosynthesis
PEP
Leucine
2-ethyl-Hexanoic acid
Pyruvic acid
Hexanoic acid
Alanine
Asparagine
Dodecanoic acid
4-Hydroxy-
phenylpyruvate
Tryptophan
Tyrosine
2-Methyl-
malic acid
Tetradecanoic acid
Lysine
Acetyl-CoA
Asparatic acid
Lactic acid
Hexadecanoic acid
HomoCys
O-Succinyl-
homoserine
Malonate
Homoserine
Methionine
Octadecanoic acid
Malate
Oxaloacetate
Citrate
Eicosanoic acid
cis-Aconitate
Nonanoic acid
TCA cycle
Fumarate
Heptadecanoic acid
Uracil
Isocitrate
Pyroglutamic acid
2-Oxo-glutaric acid
Isoleucine
Threonine
Glutamine
S-Adenosyl-
methionine
S-Adenosyl-
homocysteine
Succinate
Succinyl-CoA
Histidine
Proline
Glutamic acid
?
4-amino-Butyric acid
Amines and Polyamines
Arginine
?
Ornithine
Butylamine
Urea cycle
?
Cadaverine
?
Citruline
n-Propylamine
Arginine-succinate
Putrescine
Agmatine
Pyrophosphate (PPi)
Phosphoric acid
Nucleobases/Nucleotides
Adenine
Uridine
AMP
Guanine
Xanthine
Orotic acid
Inosine
Thymine
Pyridine, 2-hydroxy-
Pyridine, 3-hydroxy-
Thiols
Anion
Cation
Phosphoric acid monomethyl ester
Chloride
Sodium
Malic acid, 2-methylester
Thiosulfate
Sulfate
Potassium
Adipic acid
Sulfite
Boric-acid
Nitrate
Ammonium
UN
Benzoic acid
Phosphate
Furan-2-carboxylic acid
Supplemental Figure S4. Metabolite changes between sulfide, thiosulfate and sulfur growth conditions.

## Slide 5
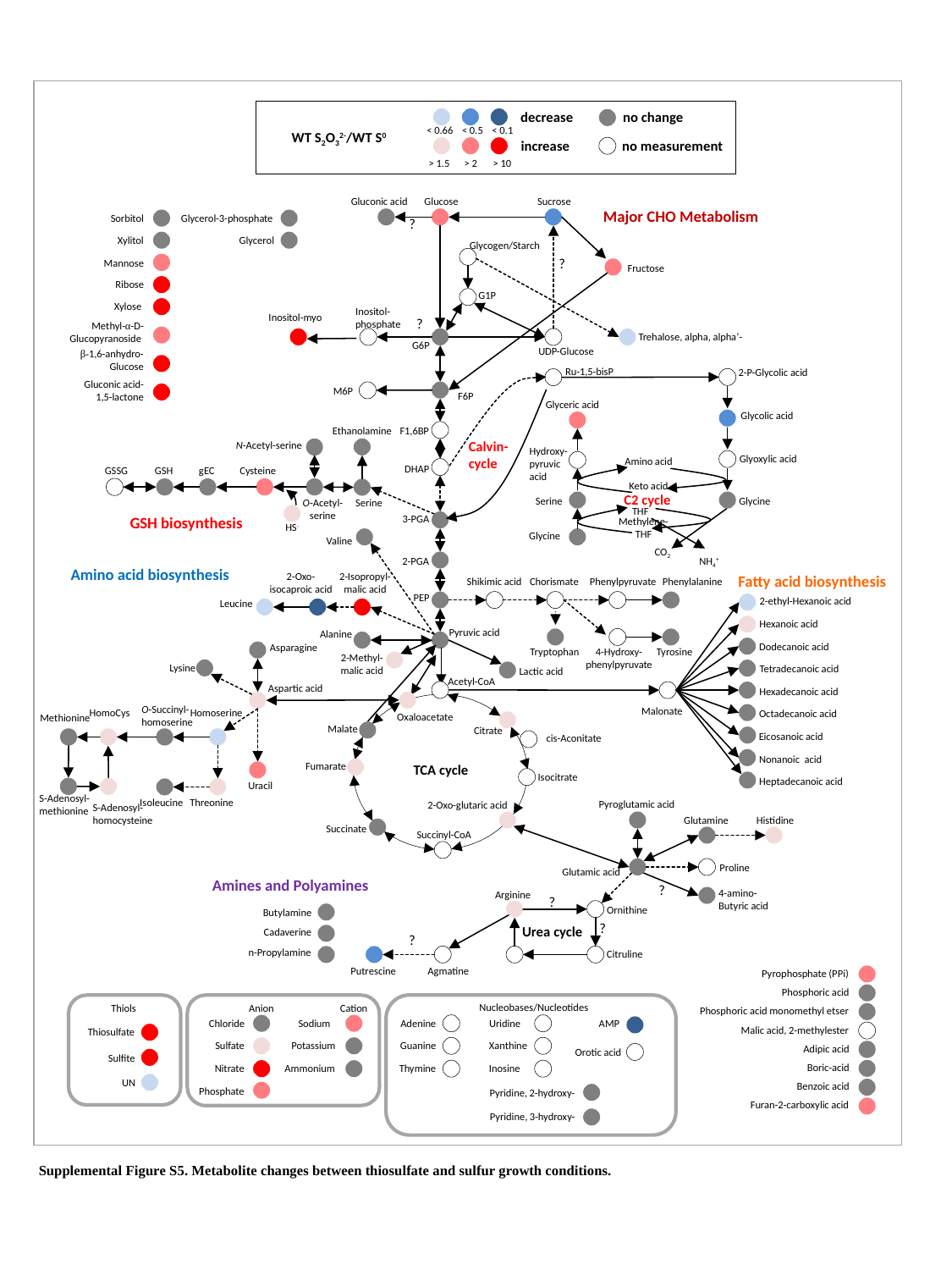

decrease
no change
< 0.66
< 0.5
< 0.1
WT S2O32-/WT S0
increase
no measurement
> 1.5
> 2
> 10
Gluconic acid
Glucose
Sucrose
Major CHO Metabolism
Sorbitol
Glycerol-3-phosphate
?
Xylitol
Glycerol
Glycogen/Starch
?
Mannose
Fructose
Ribose
G1P
Xylose
Inositol-phosphate
Inositol-myo
?
Methyl-α-D-
Glucopyranoside
Trehalose, alpha, alpha’-
G6P
UDP-Glucose
β-1,6-anhydro-
Glucose
Ru-1,5-bisP
2-P-Glycolic acid
Gluconic acid-
1,5-lactone
M6P
F6P
Glyceric acid
Glycolic acid
Ethanolamine
F1,6BP
Calvin-
cycle
N-Acetyl-serine
Hydroxy-
pyruvic
acid
Glyoxylic acid
Amino acid
DHAP
GSSG
GSH
gEC
Cysteine
Keto acid
C2 cycle
Serine
Glycine
O-Acetyl-serine
Serine
THF
3-PGA
GSH biosynthesis
Methylene-
THF
HS-
Glycine
Valine
CO2
2-PGA
NH4+
Amino acid biosynthesis
2-Oxo-
isocaproic acid
2-Isopropyl-
malic acid
Fatty acid biosynthesis
Shikimic acid
Chorismate
Phenylpyruvate
Phenylalanine
PEP
2-ethyl-Hexanoic acid
Leucine
Hexanoic acid
Pyruvic acid
Alanine
Dodecanoic acid
Asparagine
Tryptophan
4-Hydroxy-
phenylpyruvate
Tyrosine
2-Methyl-
malic acid
Lysine
Tetradecanoic acid
Lactic acid
Acetyl-CoA
Aspartic acid
Hexadecanoic acid
O-Succinyl-
homoserine
Malonate
HomoCys
Homoserine
Octadecanoic acid
Oxaloacetate
Methionine
Malate
Citrate
Eicosanoic acid
cis-Aconitate
Nonanoic acid
Fumarate
TCA cycle
Isocitrate
Heptadecanoic acid
Uracil
S-Adenosyl-
methionine
Isoleucine
Threonine
Pyroglutamic acid
2-Oxo-glutaric acid
S-Adenosyl-
homocysteine
Glutamine
Histidine
Succinate
Succinyl-CoA
Proline
Glutamic acid
Amines and Polyamines
?
4-amino-Butyric acid
Arginine
?
Ornithine
Butylamine
?
Urea cycle
Cadaverine
?
n-Propylamine
Citruline
Putrescine
Agmatine
Pyrophosphate (PPi)
Phosphoric acid
Phosphoric acid monomethyl etser
Malic acid, 2-methylester
Adipic acid
Boric-acid
Benzoic acid
Furan-2-carboxylic acid
Nucleobases/Nucleotides
Thiols
Anion
Cation
AMP
Chloride
Sodium
Adenine
Uridine
Thiosulfate
Guanine
Sulfate
Potassium
Xanthine
Orotic acid
Sulfite
Inosine
Nitrate
Ammonium
Thymine
UN
Phosphate
Pyridine, 2-hydroxy-
Pyridine, 3-hydroxy-
Supplemental Figure S5. Metabolite changes between thiosulfate and sulfur growth conditions.
